# Environmental Factors Driving Spatial Heterogeneity in Desert Halophile Microbial Communities

**DOI:** 10.3389/fmicb.2020.578669

**Published:** 2020-10-20

**Authors:** Gherman Uritskiy, Adam Munn, Micah Dailey, Diego R. Gelsinger, Samantha Getsin, Alfonso Davila, P. R. McCullough, James Taylor, Jocelyne DiRuggiero

**Affiliations:** ^1^Department of Biology, Johns Hopkins University, Baltimore, MD, United States; ^2^NASA Ames Research Center, Moffett Field, CA, United States; ^3^Department of Physics and Astronomy, Johns Hopkins University, and Space Telescope Science Institute, Baltimore, MD, United States; ^4^Department of Computer Science, Johns Hopkins University, Baltimore, MD, United States; ^5^Department of Earth & Planetary Sciences, Johns Hopkins University, Baltimore, MD, United States

**Keywords:** microbiome, extremophile, desert, heterogeneity, metagenome

## Abstract

Spatial heterogeneity in microbial communities is observed in all natural ecosystems and can stem from both adaptations to local environmental conditions as well as stochastic processes. Extremophile microbial communities inhabiting evaporitic halite nodules (salt rocks) in the Atacama Desert, Chile, are a good model ecosystem for investigating factors leading to microbiome heterogeneity, due to their diverse taxonomic composition and the spatial segregation of individual nodules. We investigated the abiotic factors governing microbiome composition across different spatial scales, allowing for insight into the factors that govern halite colonization from regional desert-wide scales to micro-scales within individual nodules. We found that water availability and community drift account for microbiome assembly differently at different distance scales, with higher rates of cell dispersion at the smaller scales resulting in a more homogenous composition. This trend likely applies to other endoliths, and to non-desert communities, where dispersion between communities is limited. At the intra-nodule scales, a light availability gradient was most important in determining the distribution of microbial taxa despite intermixing by water displacement via capillary action.

## Introduction

Understanding the relationships between microbial community composition and environmental factors is key to making robust predictions of microbiome dynamics (Green et al., 2008). Factors that limit growth or survival in a given environment will have major impact on community assembly (Goldford et al., 2018; Zhao et al., 2019). Many microbial communities are nutrient-limited, meaning that the ability to utilize the available carbon sources is the strongest force of microbial selection (Mello et al., 2016). In extreme environments, however, other factors such as water availability, nitrogen, temperature, pH, or salinity might be more critical (Frossard et al., 2015; Armstrong et al., 2016; Merino et al., 2019), making such systems valuable models for investigating processes influencing microbial community assembly (Schmid et al., 2020). In desert ecosystems, in particular, water has been identified as the core deterministic factor for microbial community assembly (Crits-Christoph et al., 2013; Merino et al., 2019; Uritskiy et al., 2019a). However, relatively few studies have investigated the relative contributions of deterministic and stochastic (i.e., non-random and random) factors to community assembly over temporal or spatial scales (Caruso et al., 2011; Feeser et al., 2018; Scola et al., 2018).

Comparative microbiome studies in the Atacama Desert, Chile, provide valuable insight into factors impacting community assembly because of the extreme conditions of humidity and temperature across the desert (Cáceres et al., 2007). Microbial communities living inside halite nodules (NaCl rocks) are of particular interest due to their diverse taxonomic composition and spatial segregation (Wierzchos et al., 2006; Davila et al., 2015). Isolated inside individual nodules, these communities appear to develop largely independently from one another (Wierzchos et al., 2006; Uritskiy et al., 2019a). Despite an average annual precipitation of less than 1 mm, microbial life in these microbiomes have evolved to rely on changes in air relative humidity (RH) (Davila et al., 2008, 2015). Facing extremely dry conditions causes their composition to be particularly sensitive to changes in the environment, making them a compelling model to investigate the effects of local climate excursions on microbiome composition and function (Uritskiy et al., 2019a).

The deliquescent properties of NaCl allow it to draw on moisture from the air above 75% RH, producing small amounts of liquid brine in the interior of halite nodules, which support halite communities in the driest parts of the Atacama Desert (Wierzchos et al., 2012; Davila et al., 2020). In some locations, the proximity of the ocean and the presence of unique wind patterns result in high RH swings during the diel cycle – from 30% during the day to 90% during the night (Cereceda et al., 2008; Finstad et al., 2016). As the halite nodules dehydrate during the day, capillary action moves brine toward the surface, resulting in an overall displacement of salt, organic molecules, and possibly even live cells (Davila et al., 2008, 2013).

Because of the saturated salt conditions, the halite microbial communities are comprised of highly adapted halophiles (Robinson et al., 2015; Crits-Christoph et al., 2016; Finstad et al., 2017). The two dominant heterotrophic taxa found in this community are halophilic *Halobacteria* (members of the *Euryarchaeota*) and *Salinibacter* (members of the *Bacteroidetes*). These halophiles are salt-in strategists, meaning they import potassium ions to counteract the external osmotic pressure from sodium ions (Oren, 2008; Gunde-Cimerman et al., 2018). Other heterotrophs in the community include halophilic *Proteobacteria*, *Actinobacteria*, and *Nanohaloarchaea* – an ectoparasite of *Halobacteria* (Crits-Christoph et al., 2016; Hamm et al., 2019). The biologically available carbon in the community is fixed by several species of *Cyanobacteria* and a single green alga (Finstad et al., 2017; Uritskiy et al., 2019b). Previous characterization of the community metatranscriptome revealed that all the major community members are transcriptionally active (Uritskiy et al., 2019b; Gelsinger et al., 2020), with photosynthesis being highly prioritized in the transcriptomes of photoautotrophs. Additionally, *Halobacteria* and *Salinibacter* also showed high levels of transcription for bacteriorhodopsin and xanthorhodopsin genes, respectively; those are modified rhodopsins that in the presence of light are used to form a proton gradient and thus generate ATP (Oren, 2014; Seyedkarimi et al., 2015).

Previous research into halite microbial community heterogeneity reported that changes in the composition of halite microbiomes over regional distance scales (tens of kilometers) were linked to moisture availability (Robinson et al., 2015; Finstad et al., 2017), although the widespread distribution of a specific *Cyanobacteria* suggested inter-site dispersal along a moisture gradient (Finstad et al., 2017). One important question remains; what is the distribution of the halite community within a nodule? Our study builds on previous findings and focuses on local distance scales to investigate the deterministic and stochastic forces influencing the halite microbial community at the meter and centimeter distance scales.

## Materials and Methods

### Sampling Scheme and Scales of Diversity

**FIGURE 1:**
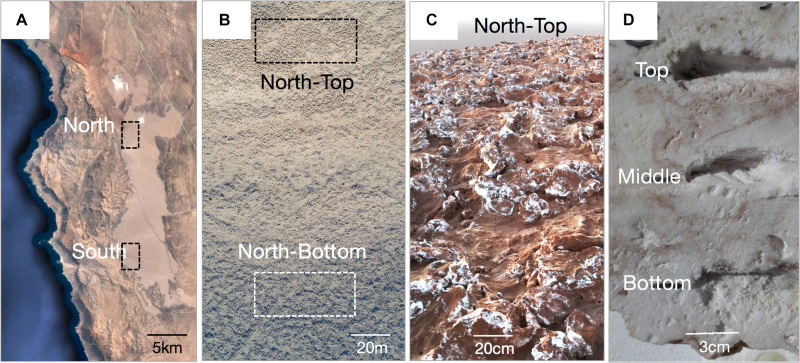
**(A)** Google Earth view of the Salar Grande with the North and South sampling regions. **(B)** Drone image of the hill at the North site showing the sampling areas at the top and bottom of the hill (photo by Mathias Meier). **(C)** Halite nodules at the top of the North hill. **(D)** Cross-section of a halite nodule with the locations of samples taken from the top, middle, and bottom of the nodule with respect to its original orientation in the field.

A total of 132 biological samples across three independent sampling efforts at the different distance scales were collected. Regional distance scales were investigated in February 2017 by sampling the North and the South ends of Salar Grande, a salar located in the Northern part of the Atacama Desert (Robinson et al., 2015; Figure 1). Samples of halite nodules were collected from 500 m^2^ areas at each end of the salar, with 39 samples from the North site and 46 samples from the South site (Supplementary Data S1, “Large Scale”). The North and South regions were 19 km apart and were at a similar distance to the Pacific Ocean (11.6 km for the North site and 9.9 km for South site). However, the height of the Coastal Mountain Range separating the salar from the Ocean was significantly different, with 450–804 m above salar level in the North and 58–205 m above salar level in the South. For more localized landscape distance scales, a hill at the North location was sampled in February 2016. The hill had 32 m of elevation gain over 330 m. 19 samples were collected from the top of the hill, and 12 from the bottom. In both sampling locations, nodules were collected in 20 m^2^ regions (Supplementary Data S1, “Medium Scale”). For the local distance scale, we performed a more detailed sampling of 6 halite nodules collected in a 10 m^2^ area at the top of the North hill in February 2018. These nodules were vertically sliced with a mechanical saw two times, separating them into three pieces. In each slice, the top, middle, and bottom sampling locations were determined by selecting three equidistant positions along the vertical axis (Supplementary Data S1, “Small Scale”). Due to the high number of samples, we harvested nodules for the different spatial scales on different dates (Supplementary Data S1), however, all direct comparisons were performed between samples collected at the same scale and on the same date. In the field, halite nodules were harvested by breaking them open with a sterilized hammer and collecting colonized pieces (1–10 cm across) from the center of the nodules in sterile Whirl-pack bags. Nodule pieces were stored in dark in dry conditions for up to 8 weeks, until further processing. Entire nodules were also wrapped in plastic, transported to the lab, and stored in similar conditions.

### Environmental Conditions

Landscape- and regional-scale atmospheric relative humidity (RH) and temperature (T) conditions were recorded for one year with HOBO Pro v2 External T/RH Data Loggers installed 1 meter above the ground at each of the three major sampling locations (North-Top, North-Bottom, and South). RH and T data were used to estimate the occurrence frequency and average duration of dew (when RH > 95%) and fog (when RH > 99%) events at each site. Intra-nodule RH and T conditions were recorded for one year with the same sensors installed inside three representative halite nodules within a 10 m^2^ area at the North-Top location. Probes were installed in the top, center, and bottom of the halite by drilling holes in close proximity to the desired position, inserting the probe, and sealing the hole back with a commercial resin. In all cases, the data loggers were set to record measurements every 30 min.

### Light Transmission Measurements Inside Halite Nodules

Light transmission in the top, middle and bottom of halite nodules was measured in controlled lab conditions (Supplementary Figure S1) with an Ocean Optics Flame-S-XR1 spectrometer (Ocean Optics, Largo, FL, United States) with a range of 220–1025 nm and equipped with a 25 μm slit and a 600 μm optical fiber probe, as previously reported (Meslier et al., 2018). In short, halite nodules were placed under controlled lighting conditions with a broad-spectrum 500 W halogen lamp 44 cm above the nodule as the only source of illumination. The optical fiber probe, equipped with a cosine corrector to homogenize the fiber optic cable’s angular response, was inserted into a tight hole drilled in the underside of the halite nodule to the desired distance from the top. The relative effective light transmission at each wavelength was estimated by dividing each measurement to the respective intensity measured from the unfiltered light source (the broad-spectrum lamp). The lamp’s spectrum was measured and used for normalization of the data to obtain the effective transmission inside the halite, thus nullifying any major differences between the spectra from the lamp and the sun. Only 500–900 nm wavelengths were considered. To account for inter-halite variability in the comparison of light transmittance to the top and middle positions of the halite nodules, the light transmittance measurements were standardized to the mean of the “top” measurements made in all three nodules. For the bottom positions within the nodules, we found that our direct transmission measurements in the lab could not reproduce realistic conditions from the desert, as light in the field scattered around and even underneath the halite nodules. The transmission measurements made at the top and middle positions inside the halite nodules were used with that of the unobstructed solar light to approximately estimate the photosynthetically active radiation (PAR) available inside the nodules. For each intra-nodule measurement, the area under the transmission curve was calculated in the 400–700 μm range, and this value was divided by that of the solar transmission spectrum to get the effective PAR reduction ratio. Because of the relatively uniform solar transmission spectrum in this range, this reduction ratio could be multiplied by the maximum observed solar PAR during midday at the sampling location (measured at 2100 μmol/m^2^/s) to obtain the approximate PAR available inside the nodules. It is important to note that the true PAR available inside the halite nodules might be somewhat higher than these estimates due to light scattering from all directions inside the pores, and additional light scattering from the sides of the nodule. To address this, an additional experiment using the same experimental set up was carried out using homogeneous 9 cm × 9 cm × 6 cm (tall) blocks of salt (American Stockman white salt, Compass Minerals, Overland Park, KS, United States). The salt block’s periphery was wrapped in aluminum foil to approximate an infinite slab of illuminated brick. Spectra were taken with the optic fiber probe aimed upward from a hole drilled from below and compared with spectra taken with the probe aimed downward in a hole at the same location in the block.

### DNA Extraction

Colonized halite pieces were ground into fine powder as previously described (Robinson et al., 2015). For intra-halite sampling, six intact nodules were vertically sliced with a mechanical saw as described above, exposing the colonization areas within. The interiors of the nodules were then scraped with a sterile knife to obtain sufficient material (2 g) and the distance to the nodule’s surface was recorded (Figure 1D). Cells were extracted from the ground halite powder as previously described (Robinson et al., 2015; Uritskiy et al., 2019a) and the DNeasy Powersoil DNA extraction kit (QIAGEN) was used to extract gDNA from the resulting cell pellet.

### Biomass Estimates by DAPI Cell Count

Total biomass in halite samples was estimated by fluorescence microscopy cell counting (Kepner and Pratt, 1994) using 0.5 g of ground halite. From each nodule vertically sliced, positions at the top, middle, and bottom (approximately equidistant) were sampled and the halite grounded into fine powder. The resulting halite powder was gradually dissolved in a solution of 20% NaCl, 1% TWEEN. The solution was gently shaken for 30 min to break cell clumps and DAPI was added to a final concentration of 0.5 μg/ml. After a 10-minute incubation, 2 ml of the solution were filtered on a 25 mm diameter black polycarbonate filter (three filter replicates in total). Filters were imaged using a DAPI (blue) fluorescent light filter at 400× magnification on a *Zeiss Imager.A1* microscope with an *X-Site series 120* fluorescence lamp illuminator. For each filter, 5 images were taken with a *Zeiss AxioCam MRm* black-and-white camera (15 images total for each halite sample; minimum field-of-view cell count was 35 cells and the mean was 364 cells). The total number of visible cells was counted in each image using an automate *CellProfiler* v2.1 pipeline, in which the *CorrectIlluminationCalculate* function was used to normalize the background light levels, and *IdentifyPrimaryObjects* function was used to find and count unique nuclei (see Supplementary Data S2 for parameters). The number of cells per gram of halite was calculated from the number of cells in each image and taking into account the *eFOV* of the camera at that magnification (0.203 mm^2^), the total area of the filter (226.98 mm^2^), and the amount of halite powder. To get a more robust cell count estimate in each biological replicate, three technical replicates were performed for each sample, and five fields of view were counted per technical replicate. Among the 15 cell count replicates for each biological replicate, replicates with estimates outside of two standard deviations of the mean were discarded.

### 16S rRNA Gene Amplicon Library Preparation and Sequencing

The 16S rRNA gene was amplified from gDNA using a 2-step amplification and barcoding PCR strategy, and primers 515F and 926R primers for the hypervariable V3-V4 region, as previously described (Needham and Fuhrman, 2016; Uritskiy et al., 2019a). PCR was done with the Phusion High-Fidelity PCR kit (New England BioLabs), the barcoded amplicons were quantified with the Qubit dsDNA HS Assay Kit (Invitrogen), pooled, and sequenced on the Illumina MiSeq platform with 250 bp paired-end reads at the Johns Hopkins Genetic Resources Core Facility (GRCF).

### 16S rRNA Gene Amplicon Sequence Variant Pre-processing

The de-multiplexed and quality trimmed 16S rRNA gene amplicon reads from the sequencer were processed with Qiime2 2018.8.0 (Bolyen et al., 2019a). The major comparison experiments (regional, landscape, and local distance scales) were processed independently. DADA2 (Callahan et al., 2016) was used to call amplicon sequence variants (ASVs) using only the forward reads of the amplicon data (options: –denoise-single, –p-trunc-len 230 –p-chimera-method consensus). Alignment MAFFT was used to create a multiple alignment of ASV sequences and phylogeny FastTree was used to construct the phylogeny tree. The sampling depth used for the core-metrics-phylogenetic generation was chosen independently in each experiment based on the sample with the lowest read coverage. For ASV taxonomy assignment, a feature-classifier was first built using the SILVA 16S rRNA gene v128 database (Quast et al., 2013) and the sequence of the 515F universal primer.

### Controls and Replication

Three samples from the top of the hill at the North sampling site were extracted twice to estimate the dissimilarity between biological replicates collected from the same original halite nodule powder sample. The corresponding six sequencing libraries were processed and sequenced the same way as the other samples in this study but sequenced on a separate run. Qiime2 beta-diversity estimation was used to estimate the average Weighted UniFrac dissimilarity between biological replicates: 0.105 ± 0.015.

### Statistical Comparisons of Community Compositions at Different Sites

All comparisons between sites were made with built-in statistical packages within Qiime2 2018.8.0 (Bolyen et al., 2019b). Alpha and beta-diversity metrics were calculated for each distance scale experiment by using the core-metrics-phylogenetic command. Alpha diversity between different sample groups was compared with the alpha-group-significance with both the PD and Evenness diversity metrics, and the significance between beta diversity between sites was computed with the beta-group-significance command using the Weighted UniFrac dissimilarity matrices (Tucker et al., 2017). The PCoA projection of the Weighted UniFrac dissimilarity matrices was imported into custom visualization scripts. Enrichment for specific taxa at each taxonomic rank was tested using the ANCOM statistical enrichment test (Mandal et al., 2015). The taxonomy of each ASV was estimated with the classify-sklearn command using a custom classifier (as described above), and the relative abundance of major taxonomic groups was imported into custom scripts for plotting and statistical analysis. Differential abundance significance of each taxon was tested using an independent two-sided *T*-test. For the intra-halite sample comparison, the relative abundances were also standardized to account for high inter-nodule and inter-slice variability. The relative abundance of each taxon in each sample was standardized to its average relative abundance in that slice. The correlation of these normalized abundances with the distance to the nodule surface was calculated with a paired sample two-sided *T*-test and fitted to a non-parametric regression with the SpatialAverage method in pyqt_fit.

## Results

### Sampling Scheme for Investigating Different Scales of Diversity

We conducted a robust sampling survey of halite nodules in Salar Grande located in the Atacama Desert, Chile. The community composition and structure were interrogated across a total of 132 biological samples in four spatial scales ranging from major regions of the salar to micro-niches within a single halite nodule (Table 1 and Figure 1). Regional distance scales were investigated by sampling the North and the South ends of Salar Grande (Figure 1A). The landscape distance scale was investigated along a hill at the North location, which had 32 m of elevation gain over 330 m (Figure 1B). For the local distance scales, we performed a more detailed sampling of six halite nodules from the top of the North hill (Figures 1C,D). These three sampling efforts (regional sampling, landscape sampling, and intra-nodule sampling) were performed in February of three separate years, and thus we process and analyze each spatial scale independently. However, it should be noted that because the region we studied is located at low latitude and at low elevation, seasonal changes are very subdued, both in terms of temperature and water availability. The interannual variability in the hyperarid core is also typically small, except for unusual rain events and wetter periods, which are often associated to el Niño periods (McKay et al., 2003), none of which were recorded during the study.

**TABLE 1:** Overview of the study design.

Scale	Distances	Distances	Condition differences
Regional	North and South sides of the salar	∼20 km	Microbiomes subject to different climate regimes
Landscape	Top and bottom of a hill	∼300 m	Microbiomes subject to slightly different local climates
Local	Inter-halite differences at the same site	∼10 m	Microbiomes in segregated and structurally unique nodules
Community	Intra-halite differences in the same nodule	∼10 cm	Micro-niches in the same halite, with varying light transmission patterns
			depending on position

### Differences in Temperature, Relative Humidity, and Light Availability Across Spatial Scales

Climate conditions in the North and South sites were significantly different. During the tested period, the South site was consistently cooler (by 5.2°C on average; Supplementary Figure S2A) and more humid (by 11% RH on average; Supplementary Figure S2B) than the North. These differences were minimal during the night and early morning. After coastal winds picked up around noon, the differences in temperature and atmospheric RH began to increase, with the greatest differences at ∼2 pm, with changes as high as 7°C and 15% for temperature and RH, respectively. Air sensors at the South site recorded 147 days with dew formation (average duration: 3 h; maximum duration: 9.5 h) and 31 days with fog formation (average duration: 9 h; maximum duration: 15.5 h). Air sensors at the North site recorded 66 days with dew formation (average duration: 2 h; maximum duration: 9.5 h) and 15 days with fog formation (average duration: 6.4 h; maximum duration: 11 h).

At the landscape scale (North-top and North-bottom) there were small differences in temperature and significant differences in moisture conditions. Specifically, the hilltop was somewhat cooler (2–3°C; Supplementary Figure S2C) and more humid (3–7%; Supplementary Figure S1D) during the morning hours. Sensors at the hilltop site recorded 85 dew days (average duration: 2.1 h; maximum duration: 8 h) and 20 fog days (average duration: 7.1 h; maximum duration: 11 h), whereas sensors at the hill bottom site recorded 47 dew days (average duration: 2 h; maximum duration: 6 h) and 10 fog days (average duration: 5.7 h; maximum duration: 8 h).

**FIGURE 2:**
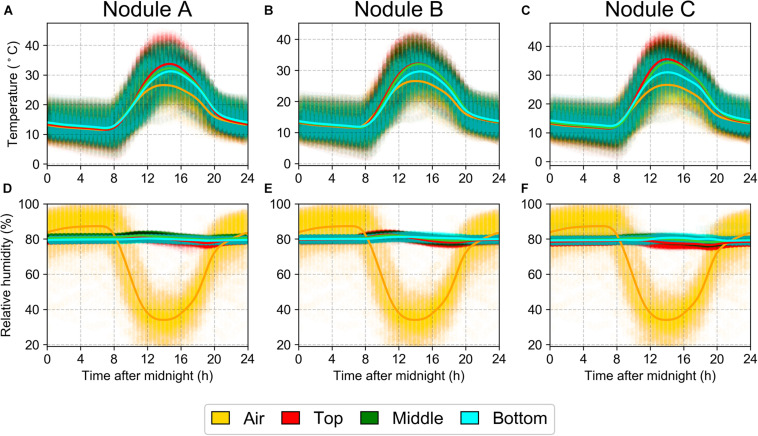
Daily average over a year (March 25, 2019 to March 11, 2020) of temperature **(A–C)** and relative humidity **(D–F)** at North-Top sampling location inside three halite nodules (columns). Data were collected with separate HOBO sensors 1m above the ground (“Air”) or sealed inside a halite nodule near the top, middle, and bottom of the nodule. Highlight lines represent non-parametric polynomial kernel regression (*q* = 6) with pyqt_fit.

The interior temperature of the halite closely tracked that of the outside air, with the temperatures near the surface of the halite sometimes reaching as high as 40°C (Figures 2A–C). The RH inside the halite nodule was notably higher than the surrounding air (Figures 2D–F), particularly during the day, when the atmospheric RH dropped significantly to as low as 20%, while the internal nodule RH never dropped below 75%. These results were reproducible across three replicate halite nodules measured at the North location. These internal nodule condition measurements were recorded one year after the intra-nodule sampling took place, however, the atmospheric temperature and RH conditions were very similar between the 2 years (Supplementary Figure S3).

A fiber optic spectrometer was used to measure transmission spectra at the top and middle positions within three independent halite nodules (Supplementary Figure S4). These spectra, together with the photosynthetically active radiation (PAR) of sunlight at its midday maximum of 2100 μmol/m^2^/s ± 105, as measured with a commercial Hobo meter, were used to calculate an average for the effective PAR available in the top- (4.70 μmol/m^2^/s ± 1.07) and middle-position (0.11 μmol/m^2^/s ± 0.06) of replicate halite nodules. The difference in available light for photosynthesis was even greater in the primary excitation wavelength of chlorophyll *a* (680 nm), with the center of the nodules receiving as little as 1% of the light usable for photosynthesis at this wavelength compared to that of the top of the nodule. To determine whether the light was approximately isotropic inside the halite nodules, transmission spectra were taken using homogenous salt blocks with the fiber optic probe positioned looking toward or away from the incident light. The light intensity inside the salt block measured with the probe aimed downward (away from the incident light) was typically 30% of the intensity at the same location but with the probe aimed upward (toward the incident light), indicating that a small correction factor of 1.3 may be applied to the measurements in halite nodules.

### Biomass Distribution Inside Halite Nodules

As an estimate for total biomass, cell numbers per g of substrate were counted for six of the nodules at the top, middle, and bottom positions (108 samples in total). Cell numbers ranged from 0.5 × 10^6^ to 9 × 10^6^ cells per gram of ground halite (Supplementary Figure S5) and there was not clear trend in biomass distribution between all the samples analyzed (Supplementary Figure S6).

### Microbial Community Structure Diversity Across Different Distance Scales

**FIGURE 3:**
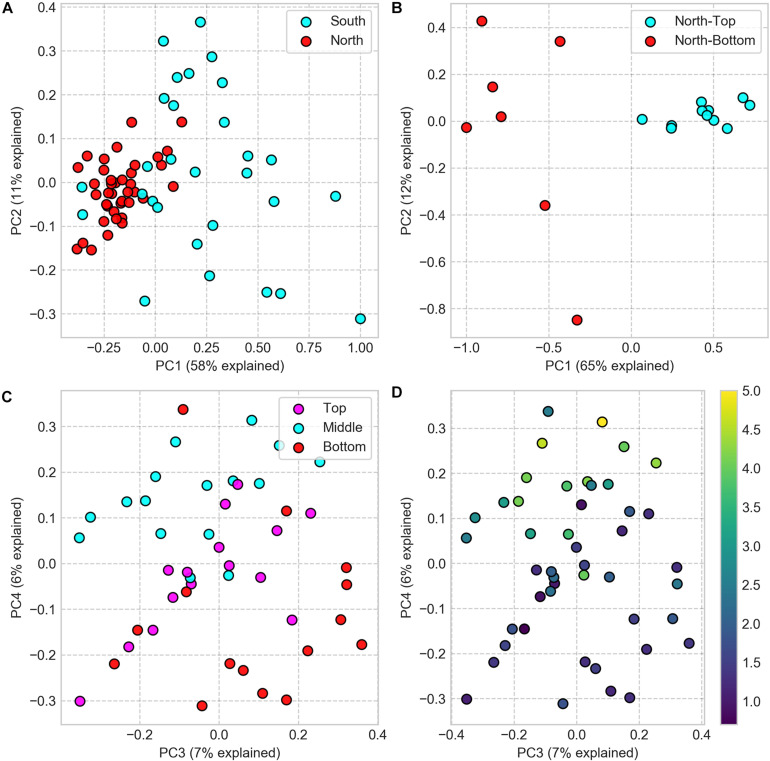
PCoA of Weighted UniFrac dissimilarity matrix of 16S rRNA gene amplicon sequences, comparing community compositions in samples from different sampling locations. **(A)** Samples from North and South sites (difference is significant, PERMANOVA: *p* < 0.001, test statistic = 28.36). **(B)** Samples from North-top and North-bottom (difference is significant, PERMANOVA: *p* = 0.001, test statistic = 22.5). **(C)** Samples from the top, middle, and bottom positions within the halite nodules (note that the scatter plot projections show the third and fourth principal components). **(D)** Same as **panel C** but colored by the sample’s distance to the nodule’s surface (distance in cm encoded in colormap).

We compared the microbial community composition between sampling sites by clustering 16S rRNA gene sequences at the amplicon sequence variant (ASV) level and by comparing each pair of samples with the Weighted UniFrac dissimilarity index, a diversity metric that measures the dissimilarity in ASV composition while accounting for ASV phylogenetic similarity. The dissimilarity matrix, resulting from this analysis, was then used to compute differences between sampling locations for all spatial scales. At the regional (North vs. South) and landscape (North-top vs. North-bottom) distance scales, we found the microbial community composition to be significantly different between sites (*PERMANOVA: p* < 0.001; Figures 3A,B). We also found that the average inter-sample dissimilarity between the North and the South was higher than that between North-top and North-bottom (0.96 and 0.91, respectively, Student’s *T*-test, *p* < 0.001).

A closer look inside neighboring nodules at the North-top sampling site revealed substantial diversity in microbial community composition inside the nodules (Figure 3 and Supplementary Figure S6). Principal coordinate analysis showed that samples only weakly separated along the first and second principal components by nodule and slice identifiers and that the top-middle-bottom spatial separation was only evident along the third and fourth principal components (Figures 3C,D). The first and second principal components explained a much greater degree of inter-sample variability (49 and 11%, respectively) compared to that of the third and fourth components (7 and 6%, respectively), suggesting that more differential ASV features were linked to the slice and nodule identifiers rather than vertical positioning within the nodules.

### Differences in Phyla Relative Abundances Across the Distance Scales

To investigate the underlying reasons for the observed differences in microbial community structure between the sampled locations, we compared the relative abundance compositions at the phylum level. As previously reported, the six most abundant phyla in halite nodule microbial communities were *Euryarchaeota* (almost exclusively comprised of *Halobacteria*), *Bacteroidetes* (primarily *Salinibacter)*, *Cyanobacteria*, *Proteobacteria*, *Actinobacteria*, *Nanohaloarchaea*, and in some cases a green alga (*Dolichomastix spp*.) (Crits-Christoph et al., 2016; Uritskiy et al., 2019b).

**FIGURE 4:**
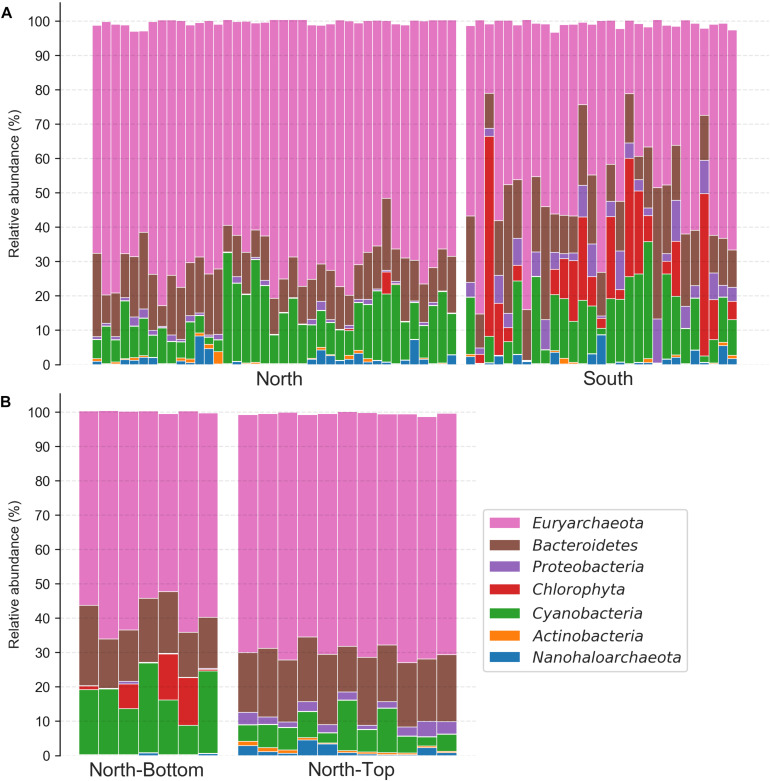
Phylum-level taxonomy composition of halite communities from different locations. **(A)** North and South ends of the salar and **(B)** the top and bottom of the North hill. Relative abundances were estimated with the Qiime2 taxonomy assignment pipeline. Only the six most abundant phyla found in this community are shown.

Focusing on these taxa, we found that the taxonomic compositions at the North and South sites of the salar differed significantly (Figure 4A and Supplementary Figure S8A), despite the high composition variability introduced by sampling over broad areas of the salar (∼500 m^2^). On average, the relative abundance of *Euryarchaeota*, which constituted the majority of the community at both locations, was higher at the North location while the relative abundances of *Chlorophyta* (a phylum of green algae) and *Proteobacteria* were higher at the South location and were almost absent in the North (Student *T*-tests, *p* < 0.0001). Unexpectedly, we found that the *Chlorophyta* (the alga’s chloroplast) 16S rRNA gene relative abundances in the South were nearly equal and sometimes greater than that of *Cyanobacteria*. Evaluating taxon differences with the analysis of the composition of microbiomes (ANCOM) enrichment test also revealed similar trends (Figure 4A). ANCOM is a differential abundance method that aims to produce few false-positives by not making any assumptions about the distribution and structure of the underlying data, and uses a W-statistic to represent the number of features that a single feature is tested to be significantly different against (Mandal et al., 2015). At the class level, *Chlorophyta* and *Gammaproteobacteria* were more relatively abundant in the South (ANCOM *W* = 20 and 18, respectively). *Cyanobacteria*, on the other hand, were significantly more relatively abundant at the North location (ANCOM *W* = 18). At the domain level, archaea (which were largely comprised of *Euryarchaeota*) were significantly more relatively abundant in the North (ANCOM, *W* = 2).

Comparing the relative taxonomic composition of halite microbial communities at the top and bottom of the North hill also revealed major differences in phyla abundances (Figure 4B and Supplementary Figure S8B). These samples were collected within 20 m^2^ areas at the top and bottom, so the inter-replicate composition variability was notably lower than that between samples collected over larger areas. *Cyanobacteria* were relatively more abundant at the bottom of the hill than at the top (Student *T*-test *p* < 0.001), while *Euryarchaeota*, *Proteobacteria*, and *Actinobacteria* were more abundant at the top (Student *T*-test *p* < 0.0001). *Chlorophyta* chloroplast sequences were only detected at low abundances in a few samples at the bottom and top, resulting in inconclusive statistical comparison. Evaluating taxa enrichment with the ANCOM significance test produced slightly different results than that from the *T*-tests on the total relative phyla abundances, specifically in the interpretation of the *Nanohaloarchaea* and *Euryarchaeota* relative abundance differences (Figure 4B). *Nanohaloarchaea* (which was not identified as differentially abundant by the *T*-test), *Proteobacteria*, and *Actinobacteria* were found to be significantly more relatively abundant at the top of the hill than the bottom (ANCOM *W* = 6, 9, 8, respectively), and *Cyanobacteria* was more relatively abundant at the bottom of the hill (ANCOM *W* = 7). No ANCOM significance in relative abundance was observed for *Euryarchaeota*, although it was significant in the *T*-test analysis.

**FIGURE 5:**
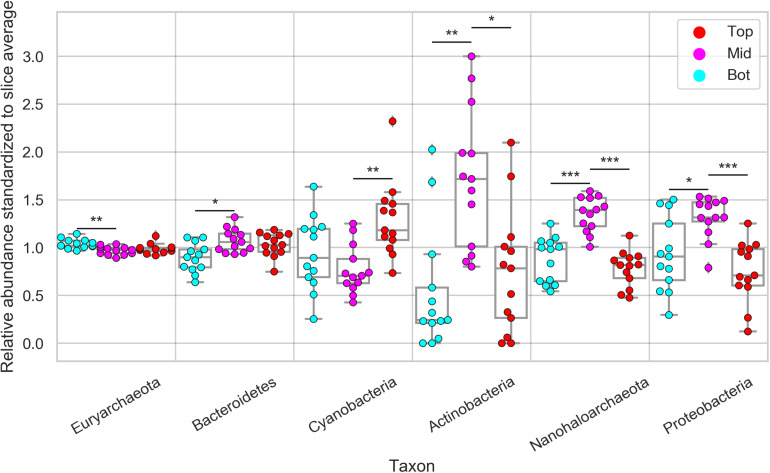
Relative abundance of major halite microbiome phyla inside the halite nodules at the top, middle, and bottom positions within each sampled nodule slice. The relative abundances were standardized to the average abundance of that phylum in each slice. Chlorophyta (chloroplast) sequences were only detected at low abundances is a few samples, and are not shown here. Bars above each phylum represent Student’s *t*-test significance, and the stars denote the associated *p*-value (***p < 0.0001, **p < 0.001, *p < 0.01).

Next, we investigated the diversity in phylum-level relative composition in different positions (top, middle, bottom) of the halite nodule interiors. Because of the high inter-nodule and inter-slice variability of the microbial community composition, the relative abundance of each taxon in each sample was standardized to its average relative abundance in that slice. This standardization resulted in a relative abundance average of 1 and highlighted differences in phyla spatial distribution along the top, middle, and bottom position of the nodules (Supplementary Figure S9). We found that *Euryarchaeota* (constituted entirely of *Halobacteria*) was more relatively abundant at the bottom of the halite than the middle, while *Bacteroidetes* showed the reverse trend, being more relatively abundant in the middle than the bottom (Student’s *T*-test, *p* < 0.01; Figure 5). However, the magnitudes of these differences were small (<8 and <20%, respectively). *Cyanobacteria* were significantly and consistently more relatively abundant by more than 80% at the tops of the nodules than the middles (Student’s *T*-test, *p* < 0.001). *Actinobacteria*, *Nanohaloarchaea*, and *Proteobacteria* were consistently more relatively abundant in the middle of the halite nodules and less relatively abundant at the top and bottom positions (Student’s *T*-test, *p* < 0.01). This preference for the center of the nodules resulted in a major increase in relative abundance at the center compared to the top and bottom positions of ∼310% for *Actinobacteria*, ∼70% for *Nanohaloarchaea*, and ∼50% for *Proteobacteria*. *Chlorophyta* (chloroplast) sequences were only detected at low relative abundances is a few samples, and thus were not included in this analysis.

We also correlated the community composition to the shortest distance of each sampling location to the surface of the nodule (either top or bottom), which confirmed preferences of some taxa toward the nodule interior versus the exterior (Supplementary Figure S9). Just as described above, the abundances of each taxon in each position were standardized to their average abundance in each slice. The significance of the positive and negative trends was evaluated with a Spearman correlation test as well as a two-sided paired *T*-test (*p* < 0.01). Similar to what was found in the categorical comparisons, *Actinobacteria*, *Nanohaloarchaea*, and *Proteobacteria* were significantly more relatively abundant further away from the surface, reaching maximum relative abundances at 2–3 cm away from the nodule surface. *Cyanobacteria* on the other hand, significantly decreased in relative abundance as the distance to the surface increased. Interestingly, both *Euryarchaeota* and *Bacteroidetes* relative abundances were not significantly correlated with the distance to the surface.

### Water Availability Dictates Community Diversity

To investigate the effects of environmental factors on community diversity, we analyzed the microbial alpha diversity between sampling locations. Comparing alpha diversity metrics across the regional and landscape scales of diversity at the ASV level revealed that the phylogenetic diversity was generally higher in the more humid locations (Supplementary Table S1). Communities from samples collected at the South site had significantly higher Faith Phylogenetic Diversity (Faith PD) than those from the North (Kruskal-Wallis, *p* < 0.001), while the Simpson and Shannon diversity indexes were not significantly different. Samples from the top of the North hill were more diverse than those at the bottom in terms of Faith PD, Shannon, and Simpson alpha diversity indexes (Kruskal-Wallis, *p* < 0.01). In contrast, at the local distance scales (intra-halite), measures of alpha diversity did not yield significant differences with categorical tests, however, a paired statistical test revealed that the center position within the halite nodules generally had higher taxonomic diversity than the top (two-sided paired *t*-test, *p* < 0.01). The other position pairings did not show a significant difference in alpha diversity (Supplementary Table S2).

### Community Structure Becomes More Similar With Increased Physical Proximity

**FIGURE 6:**
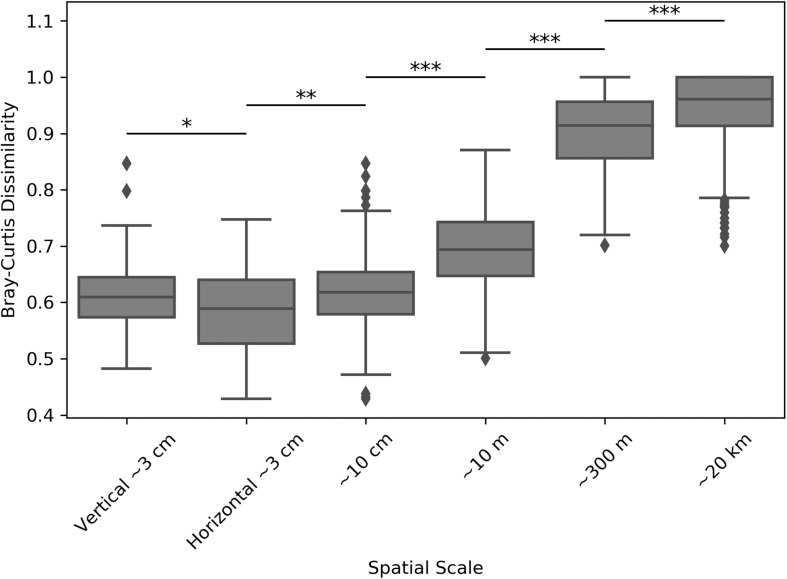
Bray-Curtis dissimilarity between microbial communities in halite samples from different sites, compared across distance scales: ∼3 cm (samples from the same nodule and position along the horizontal or vertical component), ∼10 cm (samples from the same nodule at any internal position), ∼10 m (samples from different nodules at North-top), ∼300 m (North-top vs. North-bottom), and ∼20 km (North vs. South ends of the salar). Boxplots contain the dissimilarity between all possible inter-sample comparisons in the considered sample groups. Stars denote the *p*-values of pairwise Student’s *T*-tests: ^∗^*p* < 0.05, ^∗∗^*p* < 0.01, ^∗∗∗^*p* < 0.001.

Inter-sample dissimilarity comparisons were also used to determine whether samples collected farther apart (regional distance scales) were more dissimilar than those collected closer together (smaller distance scales). Comparing average Weighted UniFrac dissimilarities between locations revealed that the average inter-sample dissimilarity was the highest at the largest distance scales, but became significantly smaller as samples became closer (Supplementary Figure S10). Performing the same analysis by using the Bray-Curtis dissimilarity metric, which highlights raw community composition differences without considering inter-ASV phylogenetic similarity, revealed an even better resolved dissimilarity differences at the tested distance scales (Figure 6). With this metric, we found that samples coming from the same positions within the halite nodules (e.g., top positions of halite 1) were more similar than those coming from different positions (Student’s *T*-tests, *p* < 0.001). In general, the inter-sample dissimilarity had a significant positive correlation with increasing distance.

## Discussion

Our detailed sampling of the halite nodule community composition across spatial scales allowed us to investigate the factors governing community assembly at each scale of diversity. Across all investigated distance scales, we found that the composition of the halite microbial communities became more similar the closer they were to one another. This finding is consistent with a previous investigation of halite microbiome diversity, where communities were more similar at the landscape distance scale compared to the regional distance scale (Finstad et al., 2017). This trend could be explained, in part, by dispersion limitation, resulting in geographic isolation and producing different community composition outcomes over time. Indeed, contained in rocks, the halite microbial communities have limited capacity to disperse, and increased distances make this even less likely. These divergent outcomes, in a process called ecological drift, are the result of stochastic composition fluctuations (Nemergut et al., 2013; Evans et al., 2017). Therefore, the observed community composition differences are likely indicative of community ecological drift, particularly at the regional distance scales where stochastic processes relating to randomized initial colonization from the seed bank (Finstad et al., 2017) can be major driving factors for community assembly (Rocha, 2018). These factors likely become less important when considering the intra-nodule distance scales where dispersion becomes less limited, as discussed below.

Deterministic factors may also play a role in shaping the halite microbial communities. Our findings showed that changes in water availability appeared to explain the observed differences in microbial community structure inside the halite nodules across all spatial scales investigated. Indeed, seemingly minor differences in atmospheric RH had significant impacts on the composition and diversity of microbial communities. Lack of dispersion between halite nodules coupled with local variations in environmental conditions appear to be sufficient to cause a divergence in community structure even at distance scales of ∼10 m.

Investigating the difference in the relative abundance of the major halite microbiome phyla across regional and landscape distance scales revealed that *Nanohaloarchaea*, *Proteobacteria*, and *Actinobacteria* were more relatively abundant in locations with higher water availability and more frequent dew and fog formation. These taxa were also consistently more abundant in the nodule centers, indicating that the nodule periphery had less favorable conditions for taxa requiring consistent water availability. While dehydration events inside the nodules were not observed during the study, the RH in the periphery of the nodules can drop below 75% during periods of prolonged dry conditions (J. DiRuggiero, pers. com). *Nanohaloarchaea*, *Proteobacteria*, and *Actinobacteria* are extremophilic that are capable of surviving long-term desiccation (Crits-Christoph et al., 2016), however, these heterotrophs are possibly less adapted than *Halobacteria* and *Bacteroidetes* to survive intermittent desiccation. Under high salt, the salt-in strategy deployed by *Halobacteria* and *Bacteroidetes* is bio-energetically more favorable than the salt-out strategy used by other halophiles (Oren, 2008; Siglioccolo et al., 2011) and could translate into a competitive advantage for surviving low water availability at saturated salt conditions. Indeed, these two phyla were more consistently abundant and diverse across regional and landscape scales of diversity. *Nanohalobacteria* are also salt-in strategists, however, they are parasitic and rely strongly on their *Halobacteria* hosts (Hamm et al., 2019). Their adaptations to a parasitic lifestyle include a small cell size and a compact and streamlined genome, both of which could diminish their adaptation to desiccation (Narasingarao et al., 2012; Crits-Christoph et al., 2016). This is consistent with a previous study investigating halite microbiome composition across regions of the Atacama Desert, where *Nanohaloarchaea* was consistently found in the more humid sampled locations (Finstad et al., 2017), and a longitudinal study showing that *Nanohaloarchaea* was less tolerant to the osmotic stress from a rare rainfall than the other taxa (Uritskiy et al., 2019a). While ecological drift could explain some of the differences in the halite community structure on regional and landscape scales, the consistent presence of certain phyla (particularly *Proteobacteria*) at the more humid locations across all scales of diversity points to water availability being the major driving factor in community assembly. This is also supported by our observations of increased alpha diversity in the microbial communities at the more humid sampling sites with more frequent dew and fog events. This is not surprising since water availability has been linked to increasing community complexity, particularly in water-limited ecosystems such as desert microbiomes (Crits-Christoph et al., 2013; Mandakovic et al., 2018), and halite nodule microbiomes in particular (Robinson et al., 2015).

Cyanobacteria was the only phylum that was more relatively abundant in the dryer sites of the salar. This is consistent with previous research across multiple salars of the Atacama Desert, where *Cyanobacteria* were found to be more relatively abundant in halite nodules of the driest salars (Robinson et al., 2015). Inside the halite interior, *Cyanobacteria* were more relatively abundant at the top of the nodules, which aligns with our light transmittance measurements showing that there was more light available for photosynthesis at the tops than at the center of the nodules. Microbial compartmentalization along the vertical axis in response to a light gradient has been well studied in microbial mats, where it was shown that phototrophic bacteria have a consistently higher relative abundance in the surface layers of the mats (Carreira et al., 2015; Nishida et al., 2018). As the light traveled deeper into the nodule, less light was available for photosynthesis, particularly at the 680 nm wavelength – the main absorbance wavelength for chlorophyll *a*. Our estimates of available PAR within the nodule interiors ranged from ∼5 μmol/m^2^/s near the top surfaces of the nodules to as little as ∼0.1 μmol/m^2^/s deep in the nodule interior, although more light could possibly reach deeper areas via vertical channel-like pores present in many nodules (see Figure 1D). Our PAR calculations are in agreement with previous experimental (Wierzchos et al., 2015) and theoretical (Nienow et al., 1988) estimates of available light in other endolithic environments.

The other oxygenic phototroph in halite microbiomes, the green alga, has been characterized with metagenomics and metatranscriptomics in the North location (Uritskiy et al., 2019b), however, our amplicon-based methods detected very low abundances at this site. In contrast, algae were very abundant at the more humid South sampling location. Previous research on these algae has shown that their relative abundance correlated with fog events (Robinson et al., 2015) and also increased after rain events (Uritskiy et al., 2019a), making the increased water availability in the South a likely explanation for their high abundances. *Halobacteria* and *Salinibacter* can also use light via microbial rhodopsins, which are light-driven proton pumps allowing them to produce chemical energy. These two taxa were found to be evenly distributed within the halite interior.

We found no consistent trend in the total number of cells at the top, middle, and bottom sections of halite nodules. While this was surprising considering the differences in light flux at these positions, these findings are also consistent with previous reports of auto-fluorescent cell counts at different positions within halite nodules from equivalently dry locations (Finstad et al., 2017). The seemingly random distribution of biomass throughout the nodules, despite deterministic factors, such a light and water, affecting the distribution of specific microbial taxa, suggests that the carrying capacity of each niche inside the nodule might be dependent on structural features of the substrate. As discussed above, the interior of the nodules is far from being homogeneous; instead, the salt matrix harbors channels, elongated pores, and cavities that are the result of salt dissolution and regular water movement (Artieda et al., 2015). This so-called architecture of the substrate, which can be difficult to quantify in halite nodules, has been reported in many other lithic microbiomes to have a great impact on endolithic microbial colonization (Walker and Pace, 2007; Meslier et al., 2018).

While we identified notable differences in community composition between the nodule core and peripheral positions, these differences were relatively subtle compared to those observed at larger distance scales. This highlights the importance of water availability as a key factor governing community composition, however, this could also imply increased rates of dispersion at the intra-nodule distance scales. A way dispersion might be increased inside nodules is via liquid water movement as a result of hydration cycles (Artieda et al., 2015). As the nodule gradually dehydrates throughout the day, water is drawn toward the surface through capillary movement; during the night, as the nodule re-hydrates, water is then draw toward the center of the nodule (Davila et al., 2008, 2013). This displacement not only drives the formation of the complex salt formations observed in the halite nodules, but might also result in the mixing of the interior microbiota over time. This idea is supported by the respective community composition at the sampled positions within the nodules, which changed predictably along the vertical component in response to a humidity gradient but changed seemingly randomly along the horizontal component. The difference in physical isolation along the two axes is possibly explained by the regular water movements between the halite core and periphery in response to the diel hydration cycles (Davila et al., 2008, 2013). This displacement of water from the inside to the outside of the nodules is also evidenced by the accumulation of scytonemin, a natural pigment produced by *Cyanobacteria*, at the surface of halite nodules (Vitek et al., 2014).

Taken together, our findings on halite microbiomes suggest that progressively smaller scales of diversity become less dependent on stochastic processes. This observation is in contrast with existing research in soil, with more evenly distributed microbiomes, where it has been reported that stochastic processes dictate assembly at smaller (centimeters) scales, while deterministic factors have the largest impact on community assembly at larger distance scales (Shi et al., 2018; Zhao et al., 2019). On the other hand, our findings are consistent within the framework of existing research in systems with non-linear segregation such as gut microbiomes. Differences in gut microbiota between individuals is driven by a combination of deterministic (e.g., diet) and neutral (stochastic colonization and community drift) processes, but differences between colonization of intestine regions are not dispersion-limited, and thus governed by deterministic factors (e.g., nutrient availability) (Albenberg et al., 2014; Li and Ma, 2016; Jha et al., 2018). Similarly, the dispersion limitation in endolithic microbiomes results in a non-linear relationship between distance and community similarity. Inter-site community variability (regional and landscape distance scales in this study) likely follows a distance-based model for community differentiation, whereby the composition of microbial communities diverges with increasing distance (Allison and Martiny, 2008; Vellend, 2010). However, our sampling scheme did not allow us to assess the relative contributions of environmental conditions and distance on the changes in microbial community composition.

In this study, we found that water, light, and community drift impact microbiome assembly differently at different distance scales, with higher rates of cell dispersion at the smaller scales resulting in a more homogenous composition. While the study focused on general taxonomic composition differences across spatial scales, differences in adaptations are likely to be more pronounced at the functional or strain level, which could be elucidated in a future shotgun metagenomics study determining what genes and pathways are enriched in response to environmental differences and investigating finer-scale taxa dispersal. Furthermore, previous research revealed that the microbial communities in separate halite nodules converge at the functional potential level but diverge at the metatranscriptomics level (Uritskiy et al., 2019b), indicating that investigating real-time transcriptional adaptations of these communities is essential to understand their functioning and adaptations to these extreme conditions.

## Data Availability Statement

The datasets presented in this study can be found in online repositories. The names of the repository/repositories and accession number(s) can be found below: https://www.ncbi.nlm.nih.gov/, PRJNA641398. All analysis scripts, intermediate data, and metadata from this study are available at https://github.com/ursky/spatial_paper.

## Author Contributions

GU, JT, and JD conceived and oversaw the study. GU, DG, AD, and JD collected in-field samples and metadata. GU, AM, MD, and SG processed field samples and constructed the sequencing libraries. PM collected and analyzed the light spectra. GU processed and analyzed the data and wrote the manuscript. AD, JT, and JD edited the manuscript. All authors contributed to the article and approved the submitted version.

## Conflict of Interest

The authors declare that the research was conducted in the absence of any commercial or financial relationships that could be construed as a potential conflict of interest.
