# Supplementary material for: Environmental Factors Driving Spatial Heterogeneity in Desert Halophile Microbial Communities
Source: Front Microbiol. 2020 Oct 20;11:578669. doi: 10.3389/fmicb.2020.578669 (PMC7606970; doi:10.3389/fmicb.2020.578669)
Supplement: Supplementary file 3 [file Data_Sheet_3.zip › Frontiers_Spatial_Supp_revised_v2.docx]

Supplementary Material

for

Environmental factors driving spatial heterogeneity in desert

halophile microbial communities

*Uritskiy et al.*

# Supplementary Data

**Data S1:** Excel file containing all collection metadata and details of environmental samples used in this study.

**Data S2:** Detailed run options used in CellProfiler for automated nuclei counting of microscopy images of DAPI-stained halite nodule samples.

# Supplementary Figures and Tables

## Supplementary Tables

**Table S1:** Pairwise Kluskal-Wallis comparison statistics between microbial community diversity at different sites, using commonly used alpha-diversity metrics.

| Alpha-diversity metric | North vs South | | Top vs Bottom | |
| --- | --- | --- | --- | --- |
|  | H | p-value | H | p-value |
| Faith_PD | 15.209357 | 0.000096 | 9.205058 | 0.002413 |
| Simpson | 0.00865 | 0.925901 | 12.157895 | 0.000489 |
| Shannon | 0.25222 | 0.615516 | 12.157895 | 0.000489 |

**Table S2:** Paired Student’s T-test comparison statistics between microbial community diversity at the top, middle, and bottom positions of halite nodules, using commonly used alpha-diversity metrics.

| Alpha-diversity metric | Top vs. Middle | | Bottom vs Middle | | Top vs Bottom | |
| --- | --- | --- | --- | --- | --- | --- |
|  | Stat | p-value | Stat | p-value | Stat | p-value |
| Faith_PD | -3.225 | 0.007 | -0.053 | 0.958 | -1.082 | 0.299 |
| Simpson | -2.701 | 0.018 | -0.564 | 0.582 | -0.601 | 0.558 |
| Shannon | -3.109 | 0.008 | -0.897 | 0.386 | -0.663 | 0.519 |

## Supplementary Figures


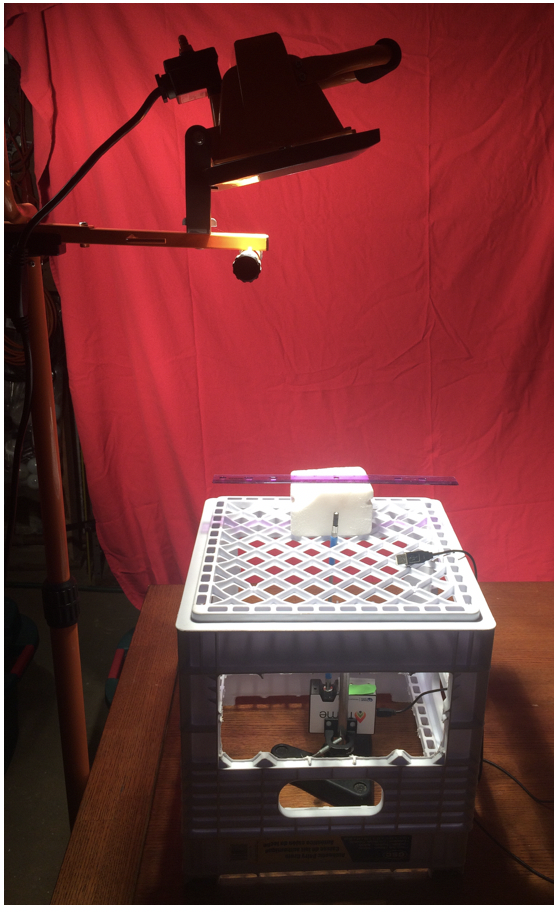


**Fig. S1:** The setup used for measuring the optical transmission of halite nodules. A 500 W incandescent lamp illuminates from above the sample supported by a milk crate. The fiber probe with the cosine corrector end piece appears in the center, with a salt block behind it, and a standard 12-inch ruler resting on it, for scale. The probe was inserted into a hole drilled into the halite or salt block and the 30-cm long fiber feeds the light to the spectrometer directly below.


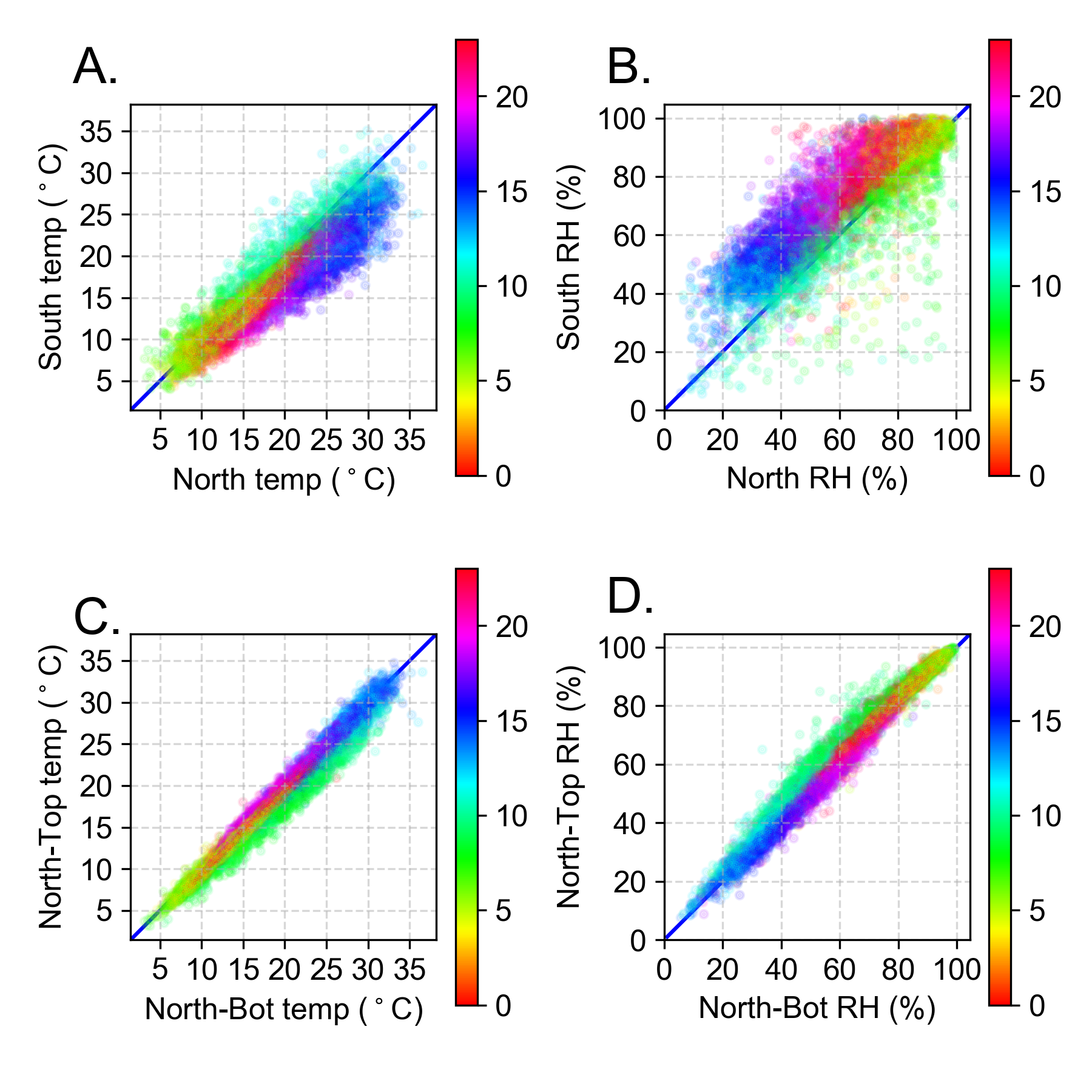


**Fig. S2:** Climate data comparisons for regional (North vs South; A, B) and landscape (top vs bottom of the North hill; C, D) scales. Weather measurements were collected simultaneously at the two compared sites, showing differences in temperature (A, C) and atmospheric relative humidity (B, D) of the locations. Colors denote the time (hours after midnight) of data collection for each point. Paired weather data was collected hourly from 2019-03-24 to 2020-03-12 with a HOBO probe located 1m above the ground.

*
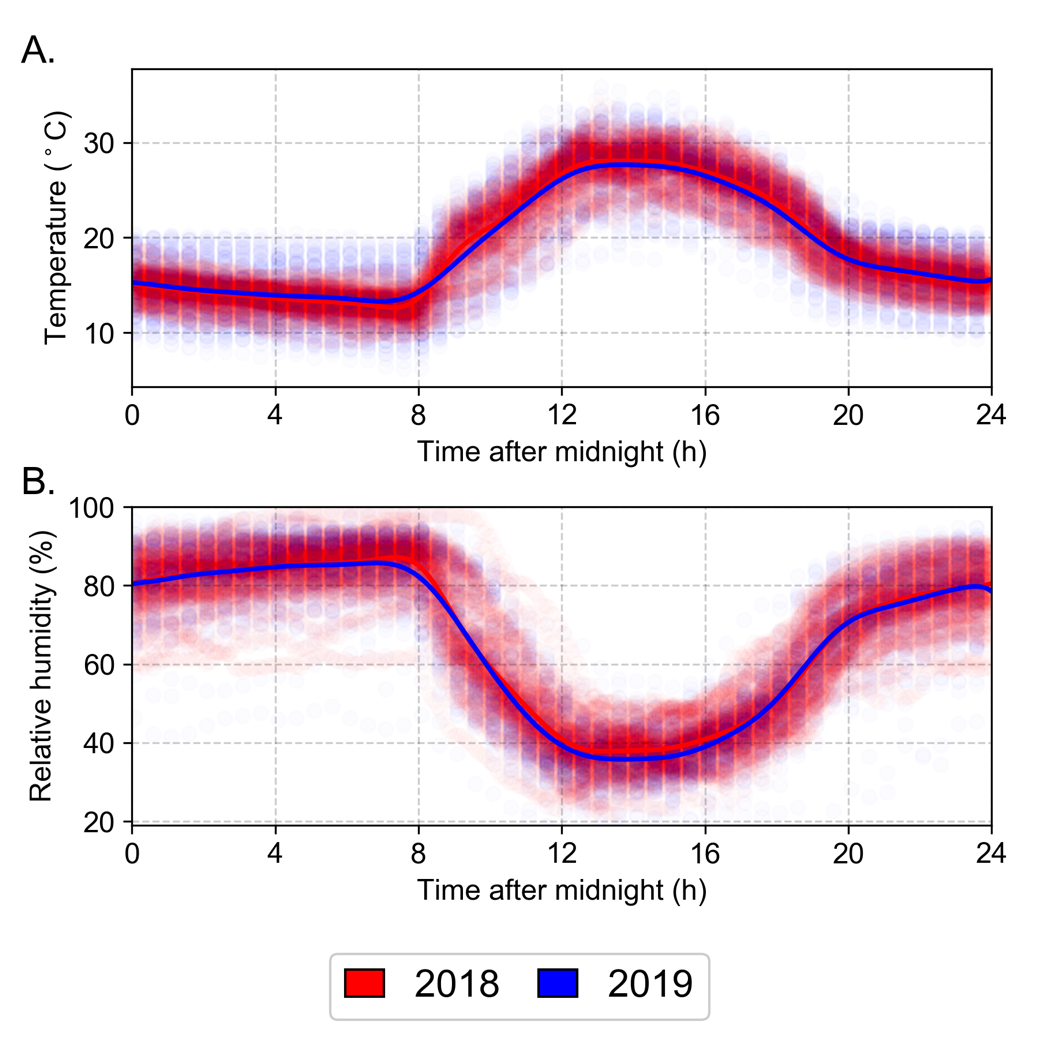
*

**Fig. S3:** Comparison in average daily temperature (A) and atmospheric relative humidity (b) between years 2018 and 2019 over four months (February – May). Data collected with the same HOBO sensor at the North-Top sampling location. Highlight lines represent non-parametric polynomial kernel regression (q=6) with pyqt_fit.


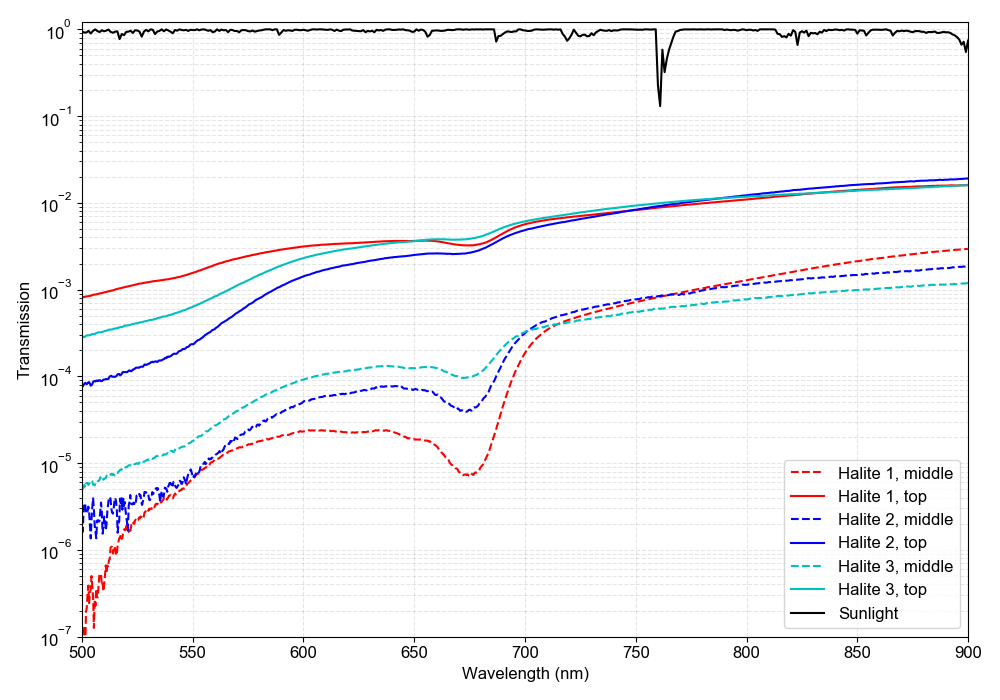


**Fig. S4:** Effective light transmission inside three halite nodules (red, cyan, and blue), measured 10 mm from the top surface (solid lines) and in the center of the nodule (dashed lines). The center positions were taken to be the middle of the three halite nodules, 30mm, 20mm, and 38mm from the surface, respectively. The transmission spectra of each nodule were normalized to the mean transmission from the top position to bring out the top-middle differences over the inter-nodule differences. The black solid line corresponds to the solar spectrum with its peak normalized to 1 on the y-axis. The photosynthetically active radiation (PAR) of this full solar during midday was measured with a Hobo PAR meter to be approximately 2100 umol/m^2^/sec, which was then used to estimate the average available PAR in the top (4.70 umol/m^2^/sec +/- 1.07) and middle nodule position (0.11 umol/m^2^/sec +/- 0.06).


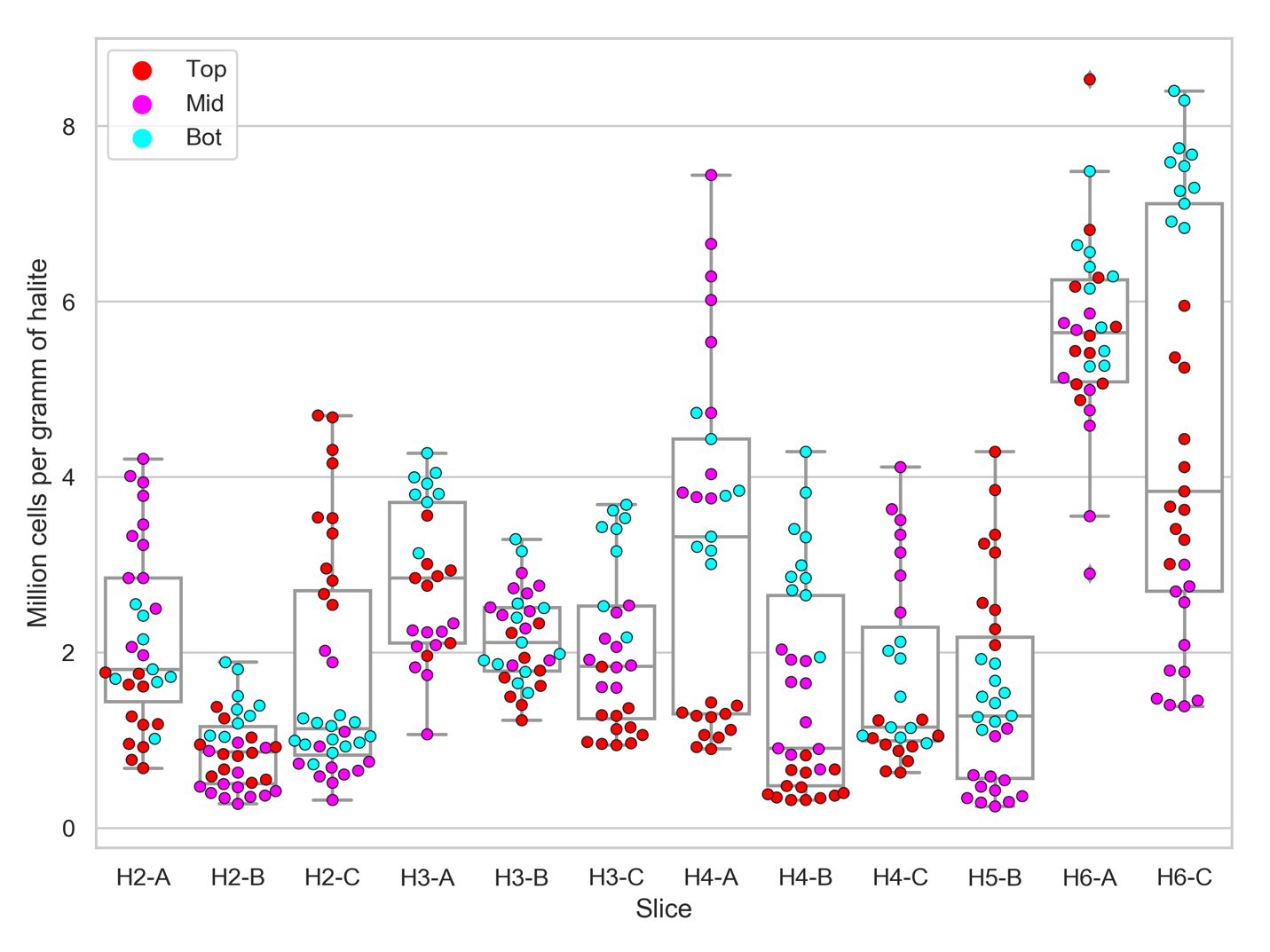


**Fig S5:** Cell numbers per g of halite within the top, middle, and bottom samples in the different halite slices. Cell counts were estimated by counting nuclei per field of view with an semi-automated cell-counting pipeline. Halite nodules are denoted H1-H6, and slices within each halite are denoted A-C. Each point represents a technical replicate from a single field of view.


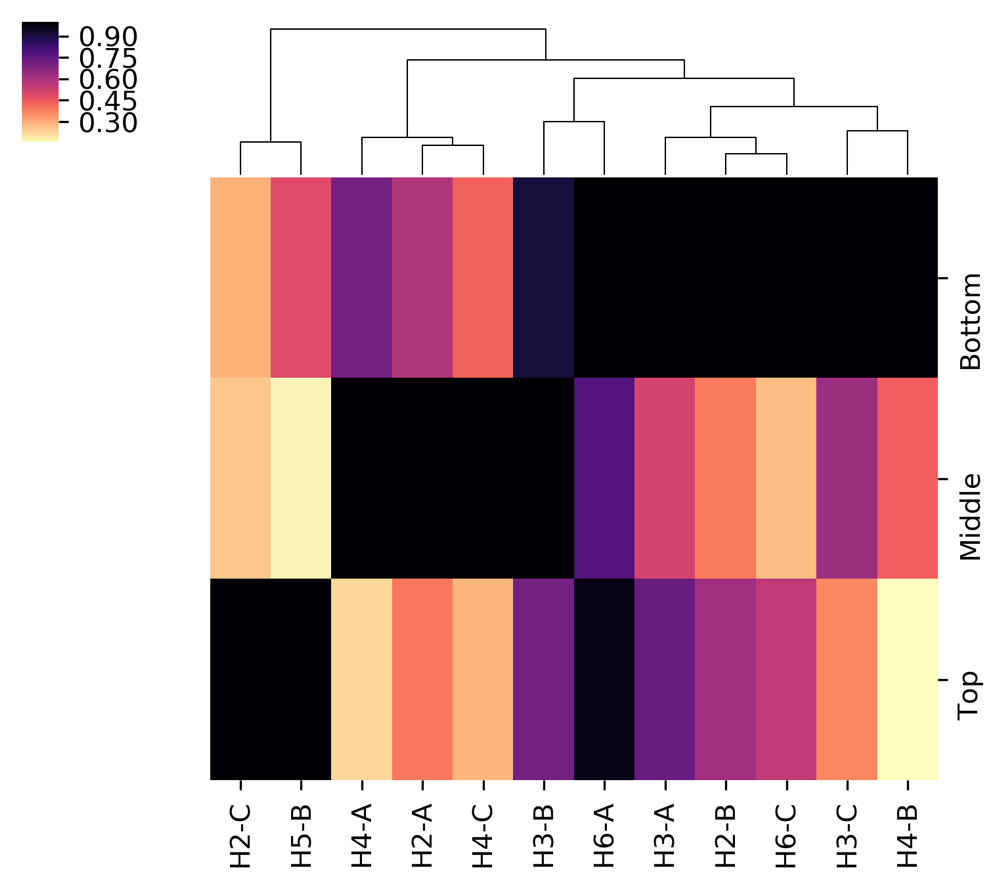


**Fig. S6:** Average cell number per g of halite for each biological sample, standardized to the maximum value within each slice (x-axis; standardized abundance encoded in color map). Black corresponds to the position with the highest cell count in that slice.

*
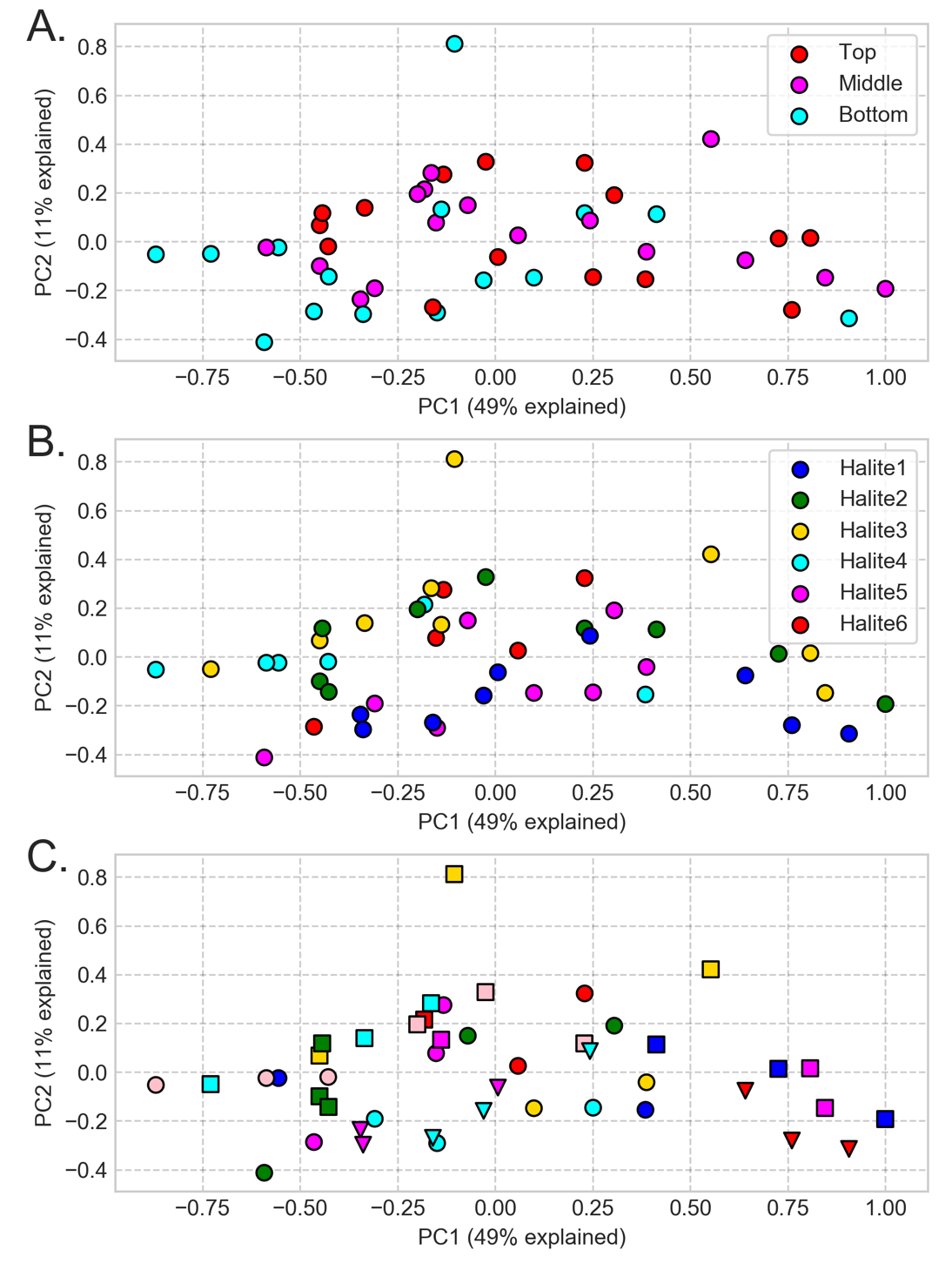
*

**Fig. S7:** PCoA of the Weighted Unifrac dissimilarity matrix of 16S rRNA gene amplicon sequences comparing community compositions in samples collected from different positions within the halite nodules. The scatterplot projections show the first and second principal components, coloring the samples by A) the samples’ relative vertical position within the halite nodules, B) the halite nodule, and C) the vertical slice of the samples’ (each slice is labeled randomly with a color-shape combination).

*
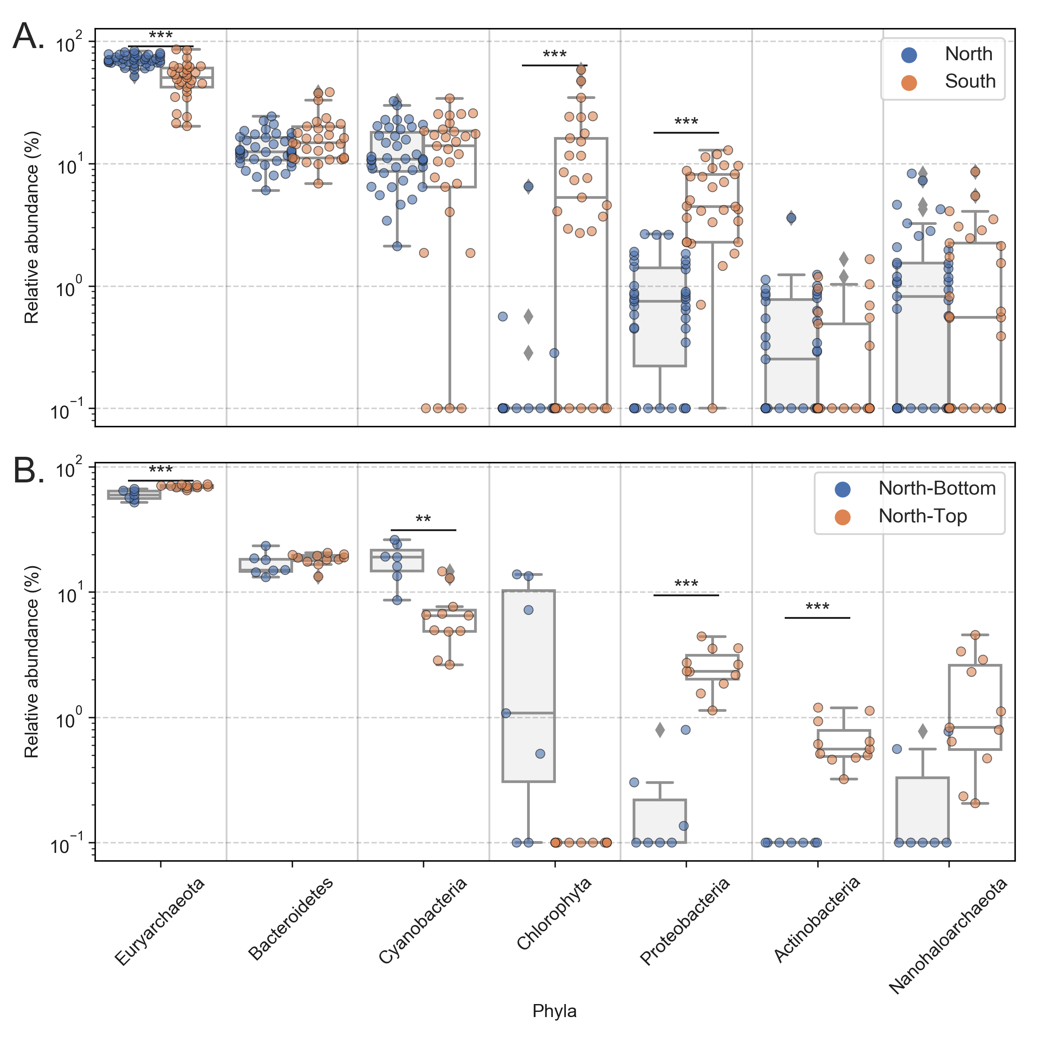
*

**Fig. S8:** Average relative abundances of the major halite microbiome phyla in samples collected in A) the North vs South and B) North-top vs North-bottom. The relative abundance of *Chlorophyta* was inferred from the relative abundance of the chloroplast 16S rRNA gene. Bars above each phyla represent Student’s t-test significance, and the star count denotes the associated p-value (***: pval<0.0001, **: pval<0.001, *: pval<0.01).

**
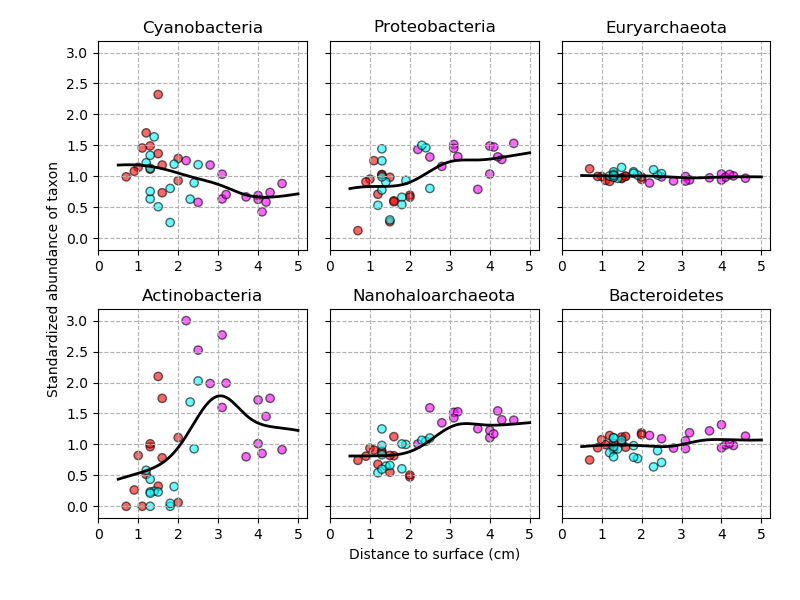
**

**Fig. S9:** Relative abundance of the six most abundant halite microbiome phyla inside the halite nodules at different shortest distances from the nodule surface (either the top or bottom), colored by relative position within the slice – red (top), middle (magenta), and bottom (cyan). The relative abundances were standardized to the average abundance of that phyla in the sampled nodule slice. The black line is the non-parametric regression of the data with the pyqt_fit package. Chlorophyta (chloroplast) sequences were only detected at low abundances is a few sample and are not shown here.


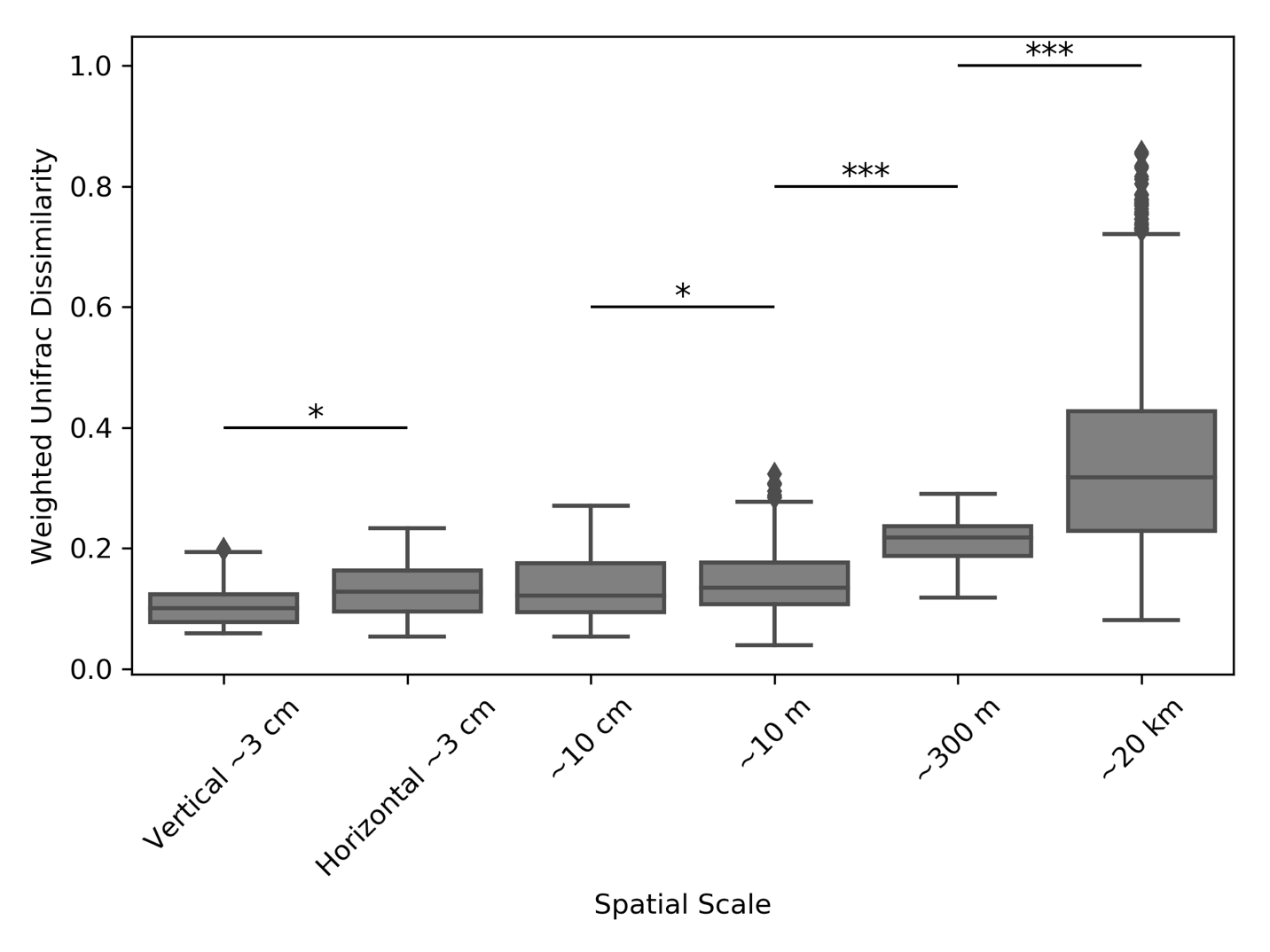


**Fig. S10:** Weighted Unifrac dissimilarity between microbial communities in halite samples from different sites, compared across different distance scales: ~3 cm (samples from the same nodule and position along the horizontal or vertical component), ~10 cm (samples from the same nodule at any internal position), ~10 m (samples from different nodules at North-top), ~300 m (North-top vs North-bottom), and ~20 km (North vs South ends of the salar). Boxplots contain the dissimilarity between all possible inter-sample comparisons in the considered sample groups. Stars denote the p-values of pairwise Student’s T-tests: p<0.05 (*), p<0.01 (**), p<0.001 (***).
